# A Fine-Tuned Positioning Algorithm for Space-Borne GNSS Timing Receivers

**DOI:** 10.3390/s20082327

**Published:** 2020-04-19

**Authors:** Xi Chen, QiHui Wei, YaFeng Zhan, TianYi Ma

**Affiliations:** 1 National Research Institute of Information Science and Technologies, Tsinghua University, Beijing 100084, China; chenxiee@tsinghua.edu.cn (X.C.); weiqh17@mails.tsinghua.edu.cn (Q.W.); 2 Department of Electronics Engineering, Tsinghua University, Beijing 100084, China; matianyi@tsinghua.edu.cn

**Keywords:** GNSS, timing receiver, space-borne, positioning algorithm, LING QIAO

## Abstract

To maximize the usage of limited transmission power and wireless spectrum, more communication satellites are adopting precise space–ground beam-forming, which poses a rigorous positioning and timing requirement of the satellite. To fulfill this requirement, a space-borne global navigation satellite system (GNSS) timing receiver with a disciplined high-performance clock is preferable. The space-borne GNSS timing receiver moves with the satellite, in contrast to its stationary counterpart on ground, making it tricky in its positioning algorithm design. Despite abundant existing positioning algorithms, there is a lack of dedicated work that systematically describes the delicate aspects of a space-borne GNSS timing receiver. Based on the experimental work of the LING QIAO (NORAD ID:40136) communication satellite’s GNSS receiver, we propose a fine-tuned positioning algorithm for space-borne GNSS timing receivers. Specifically, the proposed algorithm includes: (1) a filtering architecture that separates the estimation of satellite position and velocity from other unknowns, which allows for a first estimation of satellite position and velocity incorporating any variation of orbit dynamics; (2) a two-threshold robust cubature Kalman filter to counteract the adverse influence of measurement outliers on positioning quality; (3) Reynolds averaging inspired clock and frequency error estimation. Hardware emulation test results show that the proposed algorithm has a performance with a 3D positioning RMS error of 1.2 m, 3D velocity RMS error of 0.02 m/s and a pulse per second (PPS) RMS error of 11.8ns. Simulations with MATLAB show that it can effectively detect and dispose outliers, and further on outperforms other algorithms in comparison.

## 1. Introduction

Space-borne global navigation satellite system (GNSS) receivers are GNSS receivers carried on-board satellites, and have been used for various purposes. Space-borne GNSS receivers are primarily used for real-time orbit determination of the space vehicle [1]. In many scientific missions, the measurements of the multi-frequency on-board GNSS receivers are post-processed to achieve centimeter level orbit determination [2,3]. Space-borne GNSS receivers are also used in space-borne reflectometry for remote sensing of geophysical earth surface parameters, e.g., sea surface roughness and soil moisture [4,5,6], which has witnessed rapid development in recent years.

Space-borne GNSS timing receivers are those space-borne GNSS receivers specially designed for real time timing applications in space. The most representative users of space-borne GNSS timing receivers are low earth orbit (LEO) communication satellites. To make full use of the limited transmission power and wireless spectrum, LEO communication satellites often use smart antennas to beam-form signal power to its ground users under rapid relative space–ground movement. The prime challenge therein for designing GNSS timing receivers is how to achieve comparable positioning and precise timing performance as those stationary ground GNSS timing receivers, under a movement of several kilometers per second and at high altitudes. As far as GNSS timing receivers are concerned, commercial-off-the-shelf (COTS) GNSS timing receivers designed for low dynamic and stationary scenarios are not applicable to space applications, and those high dynamic GNSS receivers do not concern much on timing precision and robustness.

The performance of GNSS receivers, including the timing receivers, are determined mostly by its positioning algorithm. Abundant positioning algorithms have proposed in the literature over the years. Early positioning algorithms for GNSS were based on iterative least square and Kalman filtering [7,8]. Extended Kalman filtering is a natural extension to Kalman filtering for solving non-linear problems using one order linearization [9,10]. A big step forward in Kalman based filtering was the invention of unscented Kalman filter, which computes the integrals encountered in non-linear filtering problems by unscented transform [11,12]. Cubature Kalman filters (CKFs) solve the same integrals by introducing a spherical–radial cubature rule, which is mathematically more complete [13,14,15]. Robust and adaptive positioning algorithms have also been developed considering either outliers in observations or run-time parameter optimizations [16,17]. In cooperative positioning, receivers have observations not only from navigation satellites but also ranging information with wireless peers, which has led to many cooperative positioning algorithms [18,19,20].

Despite existing works, there lacks a work that systematically describes the delicate aspects of a space-borne GNSS timing receiver, specifically, how to mimic the statistical signal processing flow of a stationary ground GNSS timing receiver to achieve similar timing performance. Based on the experimental work of the LING QIAO (NORAD ID:40136) communication satellite’s GNSS receiver, we propose a fine-tuned positioning algorithm for space-borne GNSS timing receivers. Specifically, the proposed algorithm includes: (1) a filtering architecture that separates the estimation of satellite position and velocity from other unknowns, which allows for a first estimation of satellite position and velocity incorporating any variation of orbit dynamics; (2) a two-threshold robust cubature Kalman filter to counteract the adverse influence of measurement outliers on positioning quality; (3) Reynolds averaging inspired clock and frequency error estimation. Hardware emulation test results show that the proposed algorithm has a performance with a 3D positioning RMS error of 1.2m, 3D velocity RMS error of 0.02m/s and a pulse per second (PPS) RMS error of 11.8ns. Simulations with MATLAB show that it can survive typical outliers and outperforms other algorithms in comparison.The algorithm is ready to be used in the GNSS receiver of our incoming communication satellite mission.

The rest of the paper is organized as follows: Section 2 summarizes the design challenges of space-borne GNSS timing receivers, Section 3 formulates the problem and presents details of the proposed algorithm, Section 4 gives performance evaluation and Section 5 concludes the paper.

## 2. Design Challenges of Space-Borne GNSS Timing Receivers

The signal processing of a GNSS receiver can be generally divided into two steps: (1) obtain measurements from the GNSS signals; (2) calculate position, velocity and time (PVT) from the measurements using a positioning algorithm. For the positioning algorithms, while it is not hard to figure out fine PVT solutions when the measurements are abundant, it is a challenge to keep positioning precision when either the number of tracked GNSS satellites are not sufficient or the measurements have outliers. For a timing receiver, a positioning algorithm also needs to keep the one pulse per second (PPS) outputs aligning well with the start of UTC seconds under all contexts.

### 2.1. Insufficient Number of Tracked GNSS Satellites

On the ground, a stationary GNSS timing receiver is often installed at a fixed position with clear sky and enough GNSS satellites in view. With prior information of the position, such a receiver can figure out the timing when at least one GNSS satellite is available, detects anomalies when at least two GNSS satellites are available and eliminates outliers when three or more GNSS satellites are available. In contrast, a space-borne GNSS timing receiver is constantly moving. With a general Kalman positioning algorithm, it can reach a non-divergent positioning solution when at least four GNSS satellites are available, detect the existence of outliers when at least five GNSS satellites are available and find out outliers when at least six GNSS satellites are available. So when below six, the number of tracked satellites can be viewed as insufficient. Rapid spacecraft movement causes ten times bigger GNSS signal Doppler offset than that measured by ground receivers. For the sake of more Doppler search bins, space-borne GNSS receivers choose to reduce coherent integration time, thus decrease signal reception sensitivity [8,21,22]. Less sensitivity, combined with higher Earth altitude and shading caused by the spacecraft components, such as satellite-rocket detaching mechanical units, reduces the average numbers of satellites tracked by a space-borne GNSS-receiver compared to a ground-based one.

**Figure 1:**
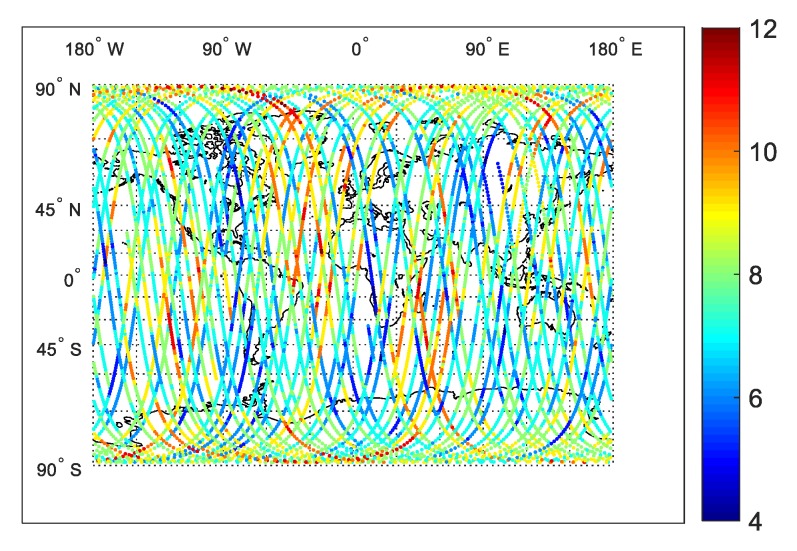
A 24 h in-orbit trace of tracked GPS satellite numbers by LING QIAO GPS receiver.

**Figure 2:**
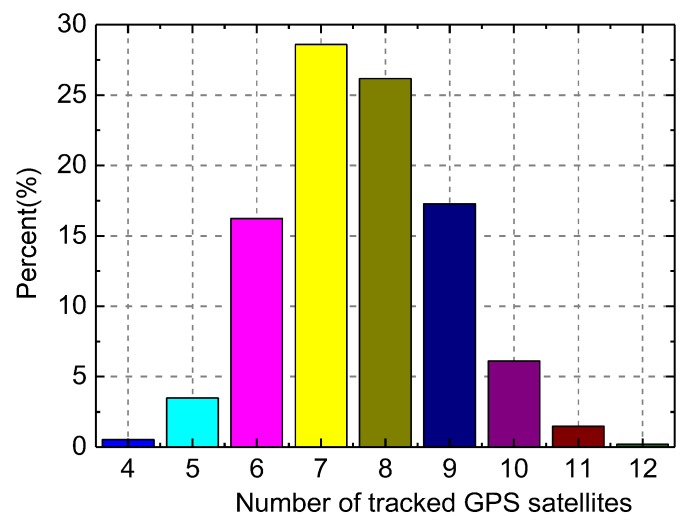
Statistical distribution of the number of tracked GPS satellites by LING QIAO global navigation satellite system (GNSS) receiver on 15 September 2017.

In Figure 1, we display a 24 h in-orbit trace of the number of GPS satellites tracked by the LING QIAO receiver on 15 September 2017. As shown, the number of tracked satellites distributes evenly in a global sense, the minimum is 4 and maximum is 12. In Figure 2, we give the corresponding statistical distribution of tracked GPS satellite numbers. As shown, the tracked satellite number is 7–8 on average, and the percent of tracked-satellite number below six also adds up to 3.9%.

### 2.2. Measurement Outliers

The space electromagnetic environment is interference-free for GNSS receivers, allowing them to track GNSS signals and obtain GNSS measurements. In most of the cases, the pseudorange error can be modeled by a Gaussian distribution with a small non-zero mean caused by GNSS satellite ephemeris and propagation path modeling errors. However, there are still measurement outliers.

**Figure 3:**
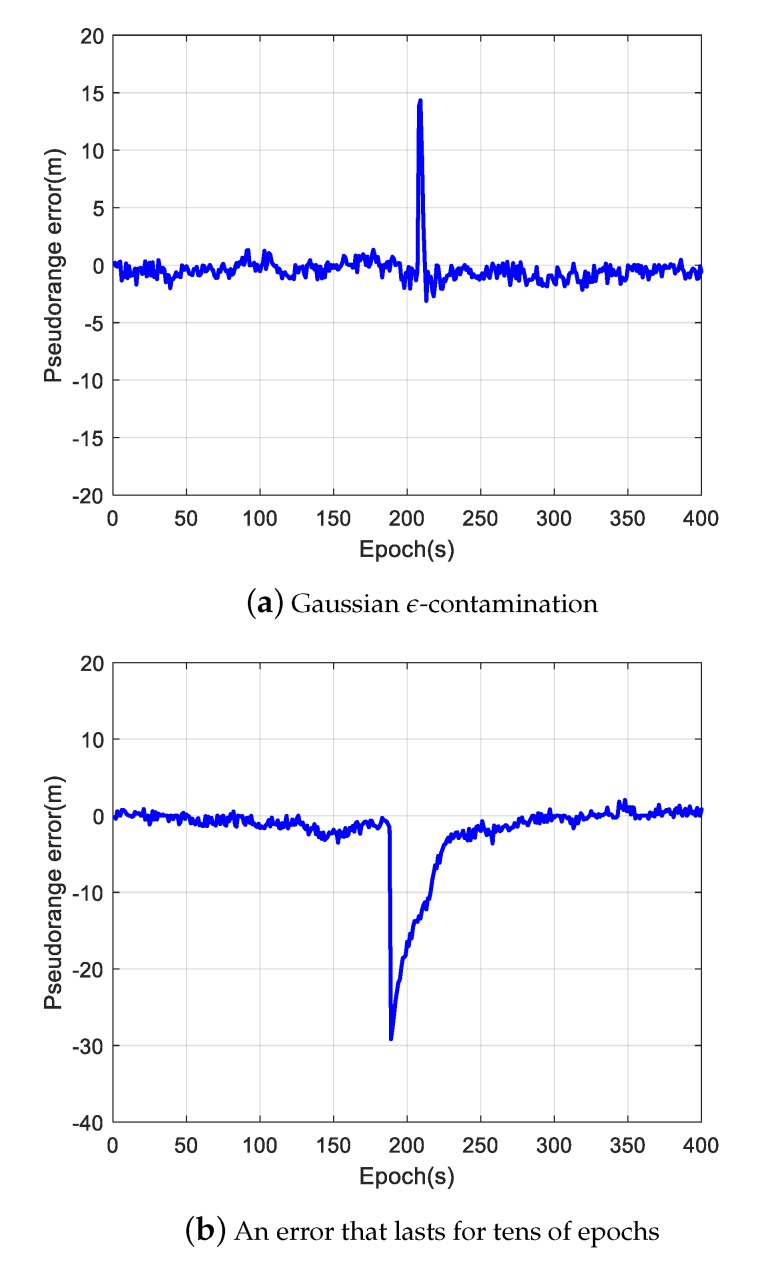

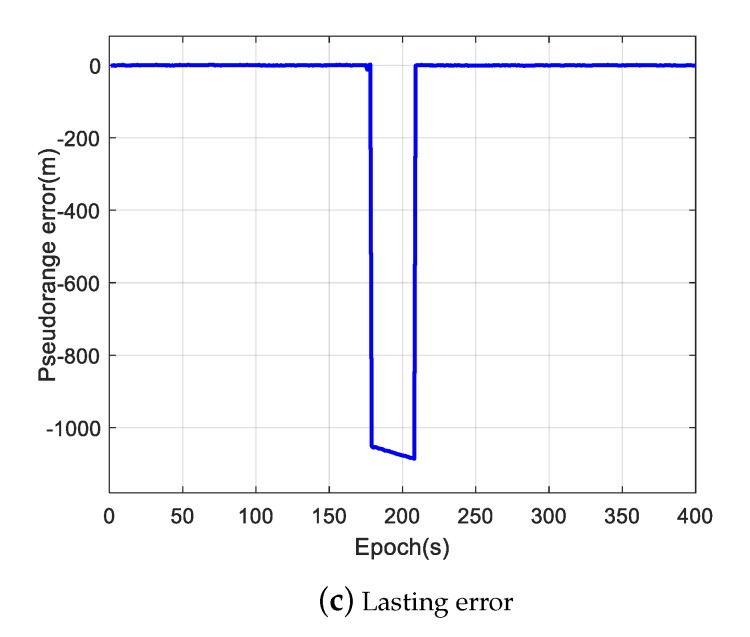
Pseudorange errors observed by the LING QIAO GNSS receiver in orbit.

Figure 3 illustrates typical pseudorange errors we found during the past four-year in-orbit operation of LING QIAO space-borne GNSS receiver. Figure 3a shows a random error, which is called Gaussian ε-contamination in robust statistics [23]. Figure 3b shows a pseudorange error that lasts for tens of epochs. The errors illustrated in Figure 3a,b are typical non-lasting outliers that are caused by many reasons such as non-perfect code tracking phase lock loop implementation, or random interference on GNSS signals. Figure 3c shows a lasting error, which is large and lasts for a period of time during the visibility of a GNSS satellite. The lasting error may be caused by wrong bit synchronization, an ephemeris error or space weather effects [24]. If not properly disposed of in GNSS receivers, lasting outlier errors would cause a maximum positioning error of several meters, which will severely influence the positioning and timing accuracy.

### 2.3. Non-Gaussian Characteristics of Clock Error

The clock error, i.e., the difference between the GNSS receiver clock and GNSS time, of a GNSS receiver is often modeled by Gaussian distribution in literals [7]. For improved timing precision, non-Gaussian characteristics of clock errors should be considered. In a GNSS receiver, the clock error is dominated by receiver clock drift, so it accumulates slowly. When the accumulation exceeds half of a receiver clock cycle, the measurement epoch should adjust by adding/subtracting one clock cycle to minimize the local clock error. The adjustment is reactive and determined by the positioning algorithm. It causes a “saw tooth” characteristic of the receiver clock error, that distributes more like a uniform distribution instead of a Gaussian distribution.

## 3. The Proposed Algorithm

### 3.1. Architecture

**Figure 4:**
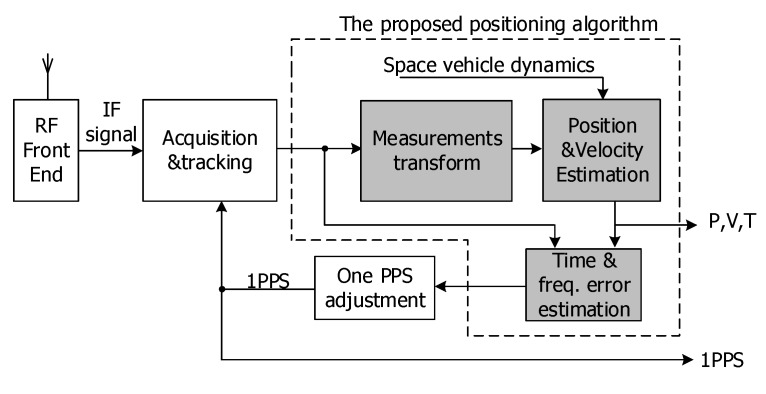
Signal processing flow of a space-borne GNSS receiver with the proposed algorithm.

As per the aforementioned observations, we propose a fine-tuned positioning algorithm for space-borne GNSS timing receivers. Figure 4 explicates the signal processing flow of a space-borne GNSS receiver with the proposed positioning algorithm. As shown in Figure 4, GNSS radio frequency signals are first down-converted to intermediate frequency signals in digital format for acquisition and tracking. The measurements of tracked GNSS satellites (see Section 3.2) are sampled at the rising edge of one PPS and being fed to the proposed positioning algorithm. The output of the proposed algorithms is PVT (position, velocity, UTC) for the user, 1PPS timing error for one PPS adjustment. The proposed positioning algorithm has three major components: (1) measurement transformations; (2) position and velocity estimation; (3) time and frequency error estimation. With the measurement transformations, as is detailed in the following sections, position and velocity can be separately estimated using historical states and arbitrary complexity of spacecraft dynamics. When the position and velocity of current epoch is determined, the time and frequency error can be estimated with at least one visible GNSS satellite, which effectively mimics the signal processing process of a stationary ground GNSS timing receiver.

### 3.2. Measurement Transformations

There are two types of measurements in a stand-alone GNSS receiver, namely pseudorange and pseudorange rate. Define $r_{i,k}^{s}={\left[p_{i,x}^{s},p_{i,y}^{s},p_{i,z}^{s}\right]}_{k}^{T}$ as the position of satellite *i* at epoch *k*, where $i=1,2,3,\ldots ,n_{s}$ and *T* represents matrix transposition. Without loss of generality, we assume the epoch interval is one second. Define $r_{k}^{u}={\left[p_{x}^{u},p_{y}^{u},p_{z}^{u}\right]}_{k}^{T}$ as the receiver position and $v_{k}^{u}={\left[v_{x}^{u},v_{y}^{u},v_{z}^{u}\right]}_{k}^{T}$ as the receiver velocity, then the pseudorange can be formulated as $y_{i,k}^{p}=\left|r_{i,k}^{s}-r_{k}^{u}\right|+b_{k}^{p}+\varepsilon_{i}^{p}$ of satellite *i*, which is obtained from the satellite code phase at epoch *k*. The $b_{k}^{p}$ is the unknown receiver clock error in meters as defined above, $\varepsilon_{i}^{p}∼N\left(0;{\left(\sigma_{i}^{p}\right)}^{2}\right)$ is pseudorange noise. The pseudorange rate can be formulated as $y_{i,k}^{d}=\frac{{\left(r_{i,k}^{s}-r_{k}^{u}\right)}^{T}}{\left|r_{i,k}^{s}-r_{k}^{u}\right|}\left(v_{i,k}^{s}-v_{k}^{u}\right)+b_{k}^{d}+\varepsilon_{i}^{d}$ which is obtained from the accumulated carrier cycles $\phi_{i,k}$ of the past second. The $b_{k}^{d}$ is the unknown receiver frequency error in meters per second and $\varepsilon_{i}^{d}∼N\left(0;{\left(\sigma_{i}^{d}\right)}^{2}\right)$ is pseudorange rate noise. There are two measurement transformations in the proposed algorithm. The first measurement transformation separates position and velocity related measurement components from other unknowns. Define measurements at epoch as:

(1) $$\left\{\begin{array}{c} Y_{k}^{p}={\left[y_{1,k}^{p},y_{2,k}^{p},\cdots ,y_{n_{s},k}^{p}\right]}^{T}\\ Y_{k}^{d}={\left[y_{1,k}^{d},y_{2,k}^{d},\cdots ,y_{n_{s},k}^{d}\right]}^{T} \end{array}\right.$$

To eliminate clock and frequency error terms in Equation (1), we define a difference mapping matrix $D_{1}=\left[I,-1\right]$ as a matrix, where *I* is $\left(n_{s}-1\right)\times \left(n_{s}-1\right)$ identity matrix and 1 is a vector of ones. According to [25], *D* reads

(2) $$D=\left[\begin{array}{cc} D_{1} & 0\\ 0 & D_{1} \end{array}\right]$$

Then we end up with the first measurement transformation:

(3) $$Z_{k}=DY_{k}=D\left({\left[\begin{array}{c} |r_{1}^{s}-r^{u}|\\ \vdots \\ r_{n_{s}}^{s}-r^{u}\\ \frac{{\left(r_{1}^{s}-r^{u}\right)}^{T}}{|r_{1}^{s}-r^{u}|}\left(v_{1}^{s}-v^{u}\right)\\ \vdots \\ \frac{{\left(r_{n_{s}}^{s}-r^{u}\right)}^{T}}{|r_{n_{s}}^{s}-r^{u}|}\left(v_{n_{s}}^{s}-v^{u}\right) \end{array}\right]}_{k}+{\left[\begin{array}{c} \varepsilon_{1}^{p}\\ \vdots \\ \varepsilon_{n_{s}}^{p}\\ \varepsilon_{1}^{d}\\ \vdots \\ \varepsilon_{n_{s}}^{d} \end{array}\right]}_{k}\right)$$

and we have

(4) $$R^{D}=DRD^{T}$$

where *R* is measurement noise matrix.

The second measurement transformation concerns the transformation the accumulated carrier cycle $\phi_{i,k}$ satellite *k*, which is a measurement of the average movement of the receiver in the past second, into the instant pseudorange rate $y_{i,k}^{d}$ of satellite *i* at epoch *k* [26]:

(5) $$y_{i,k}^{d}=\lambda \left(\frac{3}{2}\phi_{i,k}-2\phi_{i,k-1}+\frac{1}{2}\phi_{i,k-2}\right)$$

where λ is the wavelength of the navigation signal carrier. This transformation is important to a space-borne GNSS receiver because it always accelerates under the force the Earth Gravity. In contrast, a ground-based receiver often simply gets $y_{i,k}^{d}=\lambda \phi_{i,k}$. This transformation is essentially a finite impulse response filter whose z-transformation is

(6) $$H\left(z\right)=\frac{1}{T_{c}}\left(1.5-2z^{-1}+0.5z^{-2}\right)$$

This z-transformation has a zero group delay in the effective band of the LEO GNSS signal Doppler power spectrum, therefore $y_{i,k}^{d}$ exactly expresses the instant pseudorange rate of satellite *i* at epoch *k* [26,27].

### 3.3. Position and Velocity Estimation

With the above transformation, we can define the state vector as

(7) $$X_{k}={\left[{\left(r_{k}^{u}\right)}^{T},{\left(v_{k}^{u}\right)}^{T}\right]}^{T}={\left[\begin{array}{c} \begin{array}{cccccc} r_{x}^{u} & r_{y}^{u} & r_{z}^{u} & v_{x}^{u} & v_{y}^{u} & v_{z}^{u} \end{array} \end{array}\right]}_{k}^{T}$$

and the measurement matrix $Z_{k}$ has been defined by Equation (3).

We define $X_{k}=f\left(X_{k-1}\right)+W_{k-1}$ as the state update function, where f(•) is the state evolution function and is $W_{k}∼N\left(0;Q\right)$ is the evolution noise. With a first order Markov assumption and $J_{2}$ gravity perturbation model, we have

(8) $$f\left(X_{k-1}\right)=\left[\begin{array}{cc} I & tI\\ 0 & I \end{array}\right]X_{k-1}+\left[\begin{array}{c} \frac{t^{2}}{2}A_{k-1}\\ tA_{k-1} \end{array}\right]$$

where *t* is epoch interval and $A_{k-1}={\left[a_{x},a_{y},a_{z}\right]}_{k-1}^{T}$ is acceleration of the space-borne GNSS receiver. According to $J_{2}$ gravity perturbation [28], we have

(9) $$A_{k-1}={\left[\begin{array}{c} -\frac{GM}{r^{3}}r_{x}^{u}\left(1+\frac{3}{2}J_{2}{\left(\frac{R_{e}}{r}\right)}^{2}\left(1-5{\left(\frac{z}{r}\right)}^{2}\right)\right)+\omega_{e}^{2}x+2\omega_{e}v_{y}\\ -\frac{GM}{r^{3}}r_{y}^{u}\left(1+\frac{3}{2}J_{2}{\left(\frac{R_{e}}{r}\right)}^{2}\left(1-5{\left(\frac{z}{r}\right)}^{2}\right)\right)+\omega_{e}^{2}y-2\omega_{e}v_{x}\\ -\frac{GM}{r^{3}}r_{z}^{u}\left(1+\frac{3}{2}J_{2}{\left(\frac{R_{e}}{r}\right)}^{2}\left(3-5{\left(\frac{z}{r}\right)}^{2}\right)\right) \end{array}\right]}_{k-1}$$

in an Earth-fixed coordinate system. where $r=|r^{u}|$ is the receiver radius to the Earth center. GM is the Earth gravity constant, $R_{e}$ is the Earth major semi-axis, $\omega_{e}$ is the rotational angular velocity of the earth.

The state $X_{k}$ can also be predicted by using more historical states, which is out of the scope of this work. Higher orders of prediction would be more accurate but also more computation-intensive.

The proposed position and velocity estimation algorithm works as follows (Steps 1, 2 and 5 are standard steps in cubature Kalman filter (CKF), and Steps 3, 4 and 6 are innovations of this work):

1. **State Initialization**. The initial state, i.e., $X_{0}$, can be obtained using the least square algorithm. Observation noise $\sigma_{i}^{d}$, $\sigma_{i}^{p}$, prediction noise *Q* and state covariance $P_{0}$ can be given via rules of thumb.
2. **State Prediction**. Assume that $p\left(X_{k-1}\right)=N\left({\hat{X}}_{k-1\left|k-1\right.},P_{k-1\left|k-\right.1}\right)$ is known, by Cholesky decomposing, and it has
  (10) $$P_{k-1|k-1}=S_{k-1|k-1}S_{k-1|k-1}^{T}$$
  By cubature transformation [13], we have
  (11) $$\left\{\begin{array}{c} X_{i,k-1|k-1}=S_{k-1|k-1}\xi_{i}+{\hat{X}}_{k-1|k-1}\\ X_{i,k|k-1}^{*}=f\left(X_{i,k-1|k-1}\right)\\ {\hat{X}}_{k|k-1}=\frac{1}{m}\sum_{i=1}^{m}X_{i,k|k-1}^{*}\\ P_{k|k-1}=\frac{1}{m}\sum_{i=1}^{m}X_{i,k|k-1}^{*}X_{i,k|k-1}^{*T}-{\hat{X}}_{k|k-1}{\hat{X}}_{k|k-1}^{T}+Q \end{array}\right.$$
  where ${\hat{X}}_{k|k-1}$ is the state prediction and $P_{k|k-1}$ is its covariance, i=1…m, m=2n.
3. **Outlier Detection**. Define:
  (12) $$\left\{\begin{array}{c} {\hat{y}}_{i,k|k-1}^{p}=h{\left({\hat{X}}_{k|k-1}^{}\right)}_{i}\\ {\hat{y}}_{i,k|k-1}^{d}=h{\left({\hat{X}}_{k|k-1}^{}\right)}_{i+n_{s}} \end{array}\right.$$
  Calculate:
  (13) $$\left\{\begin{array}{c} {\hat{b}}_{k|k-1}^{p}=median\left(\left[y_{i}^{p}-{\hat{y}}_{i,k|k-1}\right]\right)\\ {\hat{b}}_{k|k-1}^{d}=b_{k-1|k-1}^{d} \end{array}\right.$$
  (14) $$\left\{\begin{array}{c} dy_{i,k}^{p}=y_{i,k}^{p}-{\hat{y}}_{i,k|k-1}^{p}-{\hat{b}}_{k|k-1}^{p}\\ dy_{i,k}^{d}=y_{i,k}^{d}-{\hat{y}}_{i,k|k-1}^{d}-{\hat{b}}_{k|k-1}^{d} \end{array}\right.$$
  where the median() is the middle sample whose numerical value separating the higher half of a set of data samples. If there is an even number of observations, then there is no single middle value, the median is then usually defined to be the mean of the two middle values.
  With above equations, we have the outlier elimination step: the lines of observations satisfying $dy_{i,k}^{p}>c\sqrt{{\left[diag\left(R_{k}\right)\right]}_{i}}$ or $dy_{i,k}^{d}>c\sqrt{{\left[diag\left(R_{k}\right)\right]}_{i+n_{s}}}$, where *c* is a threshold constant.
4. **Outlier Suppression**. Suppress measurements as
  (15) $${\left(Y_{ck}\right)}_{i}=\left\{\begin{array}{cc} {\left(Y_{k}\right)}_{i} & {\left(\Delta Y_{k}\right)}_{i}<K{\left(R^{\frac{1}{2}}\right)}_{i}\\ sign{\left(\Delta Y_{k}\right)}_{i})K{\left(R^{\frac{1}{2}}\right)}_{i} & else \end{array}\right.$$
  where $\Delta Y_{k}=Y_{k}-h\left(X_{k|k-1}\right)$.
5. **State Update**. Cholesky decompose $P_{k\left|k-\right.1}$:
  (16) $$P_{k|k-1}=S_{k|k-1}S_{k|k-1}^{T}$$
  By cubature transformation, the observation ${\hat{Z}}_{k|k-1}$ can be predicted by:
  (17) $$\left\{\begin{array}{c} X_{i,k|k-1}=S_{k|k-1}\xi_{i}+{\hat{X}}_{k|k-1}\\ Y_{i,k|k-1}=h\left(X_{i,k|k-1}\right)\\ {\hat{Z}}_{k|k-1}=D{\hat{Y}}_{k|k-1}=\frac{1}{m}\sum_{i=1}^{m}Y_{i,k|k-1} \end{array}\right.$$
  and the covariance $P_{zz,k|k-1}$:
  (18) $$P_{zz,k|k-1}=\frac{1}{m}\sum_{i=1}^{m}Z_{i,k|k-1}Z_{i,k|k-1}^{T}-{\hat{Z}}_{k|k-1}{\hat{Z}}_{k|k-1}^{T}+R$$
  and covariance $P_{xz,k|k-1}$:
  (19) $$P_{xz,k|k-1}=\frac{1}{m}\sum_{i=1}^{m}X_{i,k|k-1}Z_{i,k|k-1}^{T}-{\hat{X}}_{k|k-1}{\hat{Z}}_{k|k-1}^{T}$$
  Calculate Kalman gain:
  (20) $$W_{k}=P_{xz,k|k-1}P_{zz,k|k-1}^{-1}$$
  Final estimation at epoch *k*:
  (21) $${\hat{x}}_{k|k}={\hat{X}}_{k|k-1}+W_{k}\left(Z_{k}-{\hat{Z}}_{k|k-1}\right)$$
  State covariance at epoch *k*:
  (22) $$P_{k|k}=P_{k|k-1}-W_{k}P_{zz,k|k-1}W_{k}^{T}$$
6. **Measurement covariance update**. Up to now, the noise covariance of observation $Y_{k}$ is set to *R*. We recommend to use the following formula to estimate the *i*-th diagonal element of *R* at run time:
  (23) $${\left(R_{k}\right)}_{i}=\left(1-\mu \right){\left(R_{k-1}\right)}_{i}+\mu {\left[{\left(Y_{ck}\right)}_{i}-{\left(h\left(X_{k-1|k-1}\right)\right)}_{i}\right]}^{2}$$

### 3.4. Time and Frequency Error Estimation

With a robust position and velocity estimation, the clock and frequency error can be subsequently estimated. From a physical point of view, the frequency error changes slowly as receiver’s local clock drifts. To estimate such physical values, Reynolds averaging is a technique widely used in subjects such as fluid dynamics [29]. In Reynolds averaging, a physical variable is viewed as the plus of an unpredictable instant fluctuation value and a predictable average. Reynolds averaging tries to average a sequence of estimations over a time period which is long enough to smooth over the fluctuations but still short enough to keep the trend. Based on the idea of Reynolds averaging, we propose the following formula to estimate the final clock and frequency error

(24) $$\left\{\begin{array}{c} b_{k|k}^{p}=LSE\left[Y_{ck}^{p}-h^{p}\left(X_{k|k}\right)\right]\\ b_{k|k}^{d}=\left(1-\eta \right)b_{k-1|k-1}^{d}+\eta LSE\left[Y_{ck}^{d}-h^{d}\left(X_{k|k}\right)\right] \end{array}\right.$$

where LSE is a least squares estimator. If the epoch interval is 1 second, the second line of Equation (24) estimates the long term average of clock errors and the first line estimates the instant fluctuation. As long as the fluctuation does not deviate far, two times as an example, from the average, it is used to adjust the one PPS. When the fluctuation is too big, then the long term average is used to adjust the one PPS. It should be noticed that trying to solely average the clock error increases the clock error because it makes the software adjustment of one PPS counters “reluctant” to follow the random walk of a physical clock.

## 4. Performance Evaluation

### 4.1. Hardware Emulation Test Results

**Figure 5:**
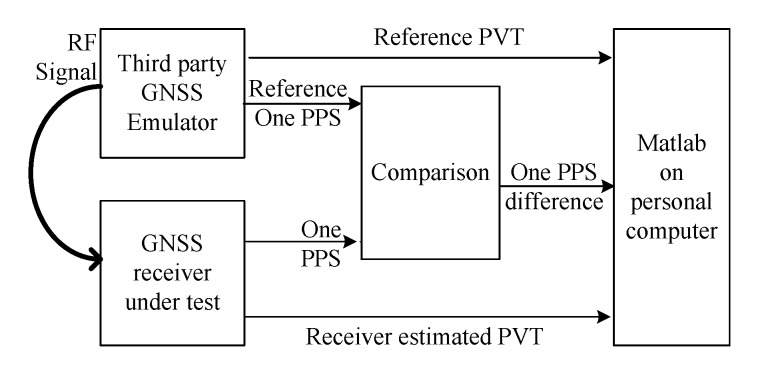
Hardware emulation setup.

**Figure 6:**
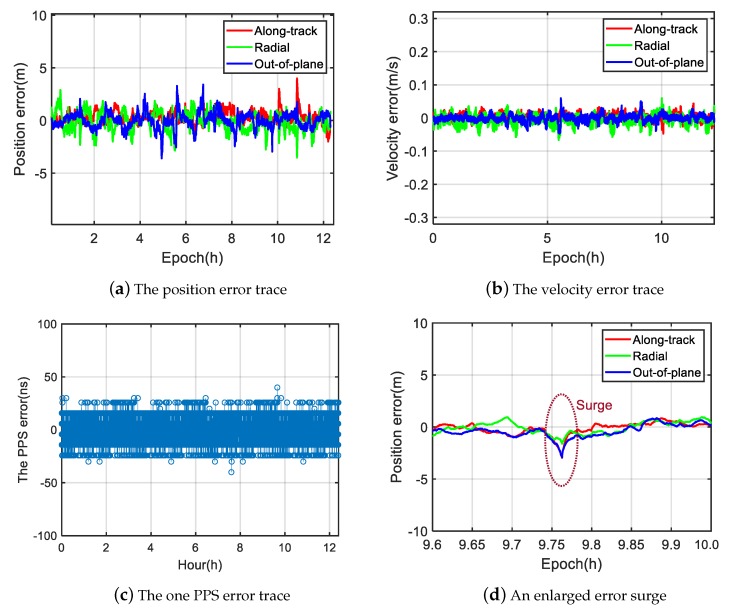
Performance of the proposed algorithm.

The hardware emulation setup is given in Figure 5. As shown, a third party GNSS emulator (GNS-8220) was used to emulate the GPS signal received at a 780 km sun synchronous orbit with a signal strength of −128 dBm. The PVT and one PPS output of an engineering GNSS receiver carried by the LING QIAO satellite were compared with the reference PVT and one PPS given by the emulator. The results were analyzed by MATLAB on a personal computer. Figure 6a shows a 12-h position error trace. The corresponding 3D position RMS error is 1.2 m. Figure 6b shows the velocity error trace, the corresponding 3D velocity RMS error is 0.02 m/s. Figure 6c shows the one PPS error trace, the corresponding RMS error is 11.8 ns.

### 4.2. Influence of Outliers

The proposed algorithm has two thresholds to minimize the influence of outliers. The first threshold is the outlier detection threshold, as defined by Step 3 of the proposed algorithm. The measurements are viewed as harmful when their predicted errors as defined by Equation (14) are larger than this threshold. Such measurements were eliminated in later steps. The second threshold is the suppression threshold as defined by Equation (15). When the predicted error of a measurement goes above the suppression threshold but below the detection threshold, it was viewed as contaminated, but can still contribute to the positioning result.

**Figure 7:**
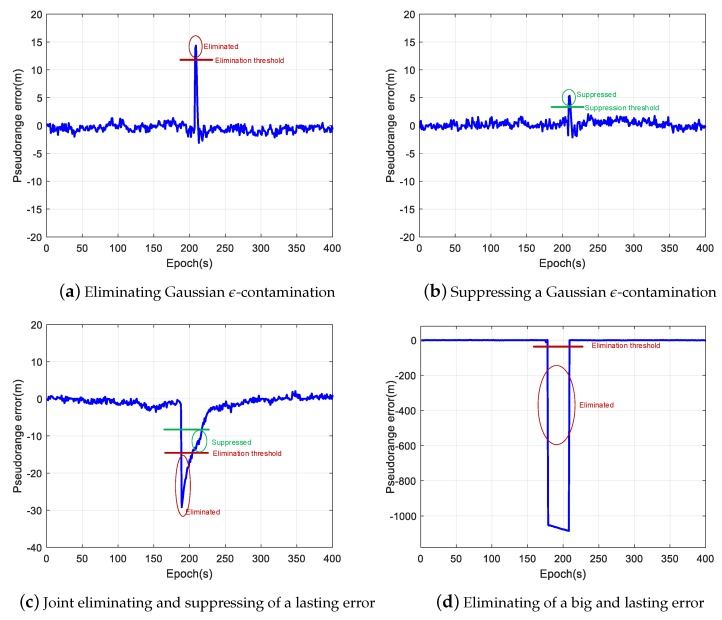
An illustration of the outliers processing of the proposed algorithm.

In Figure 7, the corresponding elimination or suppression of different outliers, as shown in Figure 3, is illustrated. If there are not enough good measurements, the suppressed outliers will still cause a surge in the positioning errors as seen in Figure 6d.

### 4.3. Comparisons with Existing Algorithms

The following two algorithms are compared with the proposed algorithm:

1. Algorithm 1: iterative least square positioning with receiver autonomous integrity monitoring (RAIM);
2. Algorithm 2: robust CKF with M-estimator [23].

Three cases were simulated. In Case 1, the MATLAB generated measurements followed an exact Gaussian distribution where $\sigma_{\varepsilon }^{p}=1$, $\sigma_{\varepsilon }^{d}=0.05$. In Case 2, the measurements were sampled during the prototyping stage of our space-borne GNSS receiver, where all typical measurement errors could be found. In Case 3, the measurements were sampled during final ground test stage, where almost all lasting system errors were removed, but there are still outliers in observations. For Algorithm 2, the $R_{k}$ is of the same Gaussian parameter as the simulated observations. For the proposed algorithm, c=10, K=3, η=μ=0.02.

The results are summarized in Table 1. In all cases, Algorithm 2 and the proposed algorithm outperforms Algorithm 1 significantly by state-based filtering. When there are outliers as in Cases 2 and 3, the proposed algorithm outperforms Algorithm 2 as it is more robust against different types of outliers.

### 4.4. Limitations

The above results were obtained by simulation or emulations. For a real GNSS receiver working in space, ephemeris error and propagation path error and other non-idealness will degrade the performance, which needs to be verified in future space missions.

## 5. Conclusions

In the past, space-borne GNSS receivers are primarily used for real-time orbit determination, or GNSS-based offline precise orbit determination, robustness is not so much a concern. For modern low earth orbit mobile communication satellites that adopt smart beam-forming to maximize data rate under limited satellite power, its on-board GNSS receiver is expected to be able to provide robust positioning and precise timing just like those stationary GNSS timing receivers widely used by ground mobile base-stations. Based on the experimental work on the GNSS receiver of the LING QIAO low earth orbit communication satellite, we proposed a positioning algorithm that is optimized for both real-time orbit determination and timing. In the proposed algorithm, measurement transformations are introduced to separate satellite position and velocity estimation from other unknowns, which allows for optimized filtering of satellite position and velocity incorporating any variation of orbit dynamics. Additionally, a two-threshold robust cubature Kalman filter was designed to counteract the adverse influence of observation outliers on position and velocity quality. After estimation of position and velocity, clock and frequency errors were estimated based on the idea of Reynolds averaging. It showed a 3D position RMS error of 1.2m, 3D velocity RMS error of 0.02m/s and a PPS RMS error of 11.8ns in hardware emulation tests. Future work includes introducing more complex space vehicle dynamics and batch based processing to smooth out errors that limits current positioning performance, network based aiding to remove GNSS satellites’ ephemeris error, etc.

## Figures and Tables

**Table 1:** Quantitative comparison of the robustness of the three algorithms.

Algorithm	Case 1		Case 2		Case 3	
	3D Pos. RMSE (m)	3D Vel. RMSE (m/s)	3D Pos. RMSE (m)	3D Vel. RMSE (m/s)	3D Pos. RMSE (m)	3D Vel. RMSE (m/s)
Algorithm 1	1.617	0.081	1.992	0.026	1.426	0.027
Algorithm 2	0.394	0.029	1.678	0.025	1.280	0.025
The proposed algorithm	0.386	0.029	1.652	0.024	1.197	0.024

## Abbreviations

The following abbreviations are used in this manuscript:

GNSS	Global Navigation Satellite Systems
LEO	Low Earth Orbit
PPS	Pulse Per Second
CKF	Cubature Kalman filter
